# Effects of yeast culture on growth performance, immune function, antioxidant capacity and hormonal profile in Mongolian ram lambs

**DOI:** 10.3389/fvets.2024.1424073

**Published:** 2024-07-23

**Authors:** Hui Chen, Shixiong Liu, Songjian Li, Dongfang Li, Xueqiang Li, Zixuan Xu, Dacheng Liu

**Affiliations:** ^1^College of Veterinary Medicine, Inner Mongolia Agricultural University, Hohhot, China; ^2^Key Laboratory of Clinical Diagnosis and Treatment Techniques for Animal Disease, Ministry of Agriculture, Hohhot, China

**Keywords:** yeast culture, production performance, immunity, antioxidant function, hormonal level

## Abstract

**Introduction:**

As effective growth-promoters and immune-modulators, yeast fermented products have shown positive effects in ruminants. To explore the mechanisms of yeast culture promoting growth and regulating immunity, this study investigated the effects of yeast culture, and β-glucan as one of its main active ingredients, on the growth performance, immune function, antioxidant capacity and hormonal profile in Mongolian ram lambs.

**Methods:**

One hundred and five Mongolian ram lambs were randomly assigned to 3 groups, with 35 replicates in each group. The dietary treatments were: total mixed ration (TMR) as the control group, TMR supplemented with 50–70 g/kg yeast culture (YC) or 75 mg/kg β-glucan. The test period was 137 days. All the sheep were weighed and 6 serum samples were collected in each group on days 0, 30, 60, 90 and 130, respectively.

**Results:**

The results showed that both YC and β-glucan could promote the growth performance with increased average daily gain and decreased feed to weight gain ratio. Moreover, these two feed additives facilitated the immune function by selectively increasing the serum levels of lysozyme, IgG, IgM, INF-γ, TNF-α and some interleukins (IL-1β, IL-2, IL-6 and IL-8); ameliorated the antioxidant capacity with higher total antioxidant capacity and enzyme activities of catalase and glutathione peroxidase; altered the metabolism-associated hormone levels with higher growth hormone and thyroid hormone T3 but lower cortisol and insulin.

**Discussion:**

In conclusion, both YC and β-glucan could improve the growth performance, immune function and antioxidant capacity, and regulate the serum levels of metabolism-associated hormones, thus exerting effects of promoting growth and improving immune function. Therefore, YC could be considered as a suitable potential alternative strategy to antibiotics and be used as an animal feed additive. This article provides a theoretical basis for the clinical application of such yeast fermented preparations in mutton sheep husbandry.

## Introduction

In mutton sheep breeding, factors such as weaning stress, sudden change of lamb diet, group transformation, vagaries of the weather and long-distance transport often cause high rates of lamb diarrhea, elimination or even death, resulting in great economic losses (1). To address these issues, various forage additives have been employed to decrease diarrhea morbidity and lamb mortality, and to promote animal growth, and among them antibiotics have been widely used in diets (2). However, the utilisition of in-feed antibiotics in animal husbandry is frequently questioned because they have been proved to lead to serious complications due to drug resistance and the presence of antibiotic residues in animal products (3). On the other hand, probiotics and their cultures are showing more and more promising effects on animal health and human well-being, and may be considered as suitable alternative strategies to antibiotics in recent years (4, 5).

It has been proved that yeast cultures (YCs) can improve ruminal fermentation, milk production and feed efficiency, as well as regulate the oxidative status and immune response of ruminants (6–8). Furthermore, YCs can ameliorate intestinal structure and stimulate intestinal development, and modify the ecological environment (9). As the main components of yeast cell wall, polysaccharides, including β-glucans and mannans, have been illustrated to play important roles in enhancing antioxidant capacity and immune response in sheep and other ruminants (10, 11). Yeast-derived β-glucan, which consists of a (1,3)-β-linked backbone and a small amount of (1,6)-β-linked side chains, is known for its immunostimulatory effects (12). In addition, it has been widely reported that β-glucan exerts obvious beneficial effects on the health and production performance of ruminants, especially on gut health, immunity, and ruminal development (13).

At present, many probiotic fermented products for ruminant animals have emerged, yet their quality and efficacy vary considerably. Yeast culture (YC) is a microbial fermentation preparation previously developed and already commercialized by our research group, fermented by two strains of highly active yeast with a defined medium through special fermentation process (14). The principal active components of the product are β-glucan, mannan, amino acids, polypeptides and organic acids, and its two main effects are to enhance immune function and digestive ability of the animals (15). The two dominant yeast strains (XR4 and BC) identified by our research group were isolated from naturally fermented milk in Inner Mongolia’s primitive grasslands and these two strains have shown significant advantages in cell biomass, enzyme production ability, and especially in the ability to secrete active substances (15). With XR4 and BC as fermentation strains, the contents of β-glucan and mannan in YC increased by 30 and 46%, respectively, after special processes such as liquid enrichment culture, solid-state fermentation, yeast wall breaking, and carrier adsorption (16). Therefore, to explore the mechanisms of YC as a potential alternative to growth-promoting and disease-preventing antibiotics, this study investigated the effects of oral administration of YC or one of its main functional factors, yeast-derived β-glucan, on the growth performance, immune function, antioxidant capacity and hormonal levels of Mongolian ram lambs.

## Materials and methods

### Preparation of yeast culture

YC is a previous research achievement of our group, which is a mixed microbial fermentation product produced by two strains of highly active yeast through liquid enrichment culture, solid anaerobic fermentation, yeast wall breaking and other processes (14, 15). Specifically, one strain of *Saccharomyces cerevisiae* (BC) and one strain of *Candida utilis* (XR4), sourced from the strain bank of the Ruminant Microecological Preparation Research Group of the School of Veterinary Medicine Inner Mongolia Agricultural University, were mixed in 1:1 ratio and injected into the solid fermentation material, with the proportion of the former being 10% of the total inoculum. The solid fermentation material consists of 20% wheat bran, 10% standardized flour, 35% corn, 7% corn protein powder, 8% rice bran meal, and 20% soybean meal. Water was added to make the water content finally reach 38% ~ 40%. The solid state stacking fermentation was performed in a laboratory setting, with a stacking height of 60–65 cm and a fermentation time of 72 h. The temperature of the material was recorded every 3 h during the fermentation process. When the fermentation time reached 24 h and the temperature reached 40°C or above, a turnover was initiated, which was followed by a continued stacking fermentation. Subsequently, the yeast culture was dried at a low temperature (45–50°C) and crushed and bagged. The main nutritional contents of YC are as follows: crude protein ≥18.0%, mannan ≥0.5%, crude ash ≤9.0%, moisture ≤12.0%, number of active yeast ≥10^6^ cfu/g.

### Animals and diet

Animal experiments were conducted by the guidelines for animal experiments of the National Institute of Animal Health of China (GB 14925-2010) and were approved by the Ethics Committee of Inner Mongolia Agricultural University (NND2021072). The study was conducted from April to August 2021 at the Mongolian mutton sheep farm in Urad Front Banner, Bayannur City, Inner Mongolia, China. The area is located at 108 ° 11 ′ E, 40 ° 28 ′ N, with an elevation of 1,023 meters and a dry climate. One hundred and five Mongolian ram lambs (age = 50 ± 5 days, BW = 12.74 ± 1.06 kg) were selected and randomly divided into three groups with 35 lambs in each group, including a control group and two treatment groups. The control group (Control) was fed a basic total mixed ration (TMR) diet. In addition, the sheep in the first treatment group were fed a TMR diet supplemented with yeast culture (YC, 50–70 g/kg), while β-glucan (BG, 75 mg/kg, produced by Angel Yeast Co., Ltd.) was added to TMR diet in the second treatment group. It is noteworthy that the diets of the three groups were prepared according to the feeding standard for mutton sheep in China (NY/T 816-2004), and the energy and nutrient levels were consistent among the 3 groups. The ingredients and chemical composition of the diets are shown in Table 1. The test period was 137 days, including 7 days of a pre-trial period and 130 days of a trial period. During the pre-trial period, all sheep were dewormed and vaccinated, the barns and pens were disinfected with the same protocol. Each sheep was housed in a separate pen, equipped with a feed trough and water dispenser. In addition, the sheep were fed twice per day, at 06:00 and 18:00, free access to water.

**Table 1:** Ingredient composition and nutrient levels of diets (dry matter basis).

Items	Pre-test period		Mid-test period		Late-test period	
	YC	Control and BG	YC	Control and BG	YC	Control and BG
Ingredients, %						
Peanut vine	22.00	22.00	13.50	14.00	10.00	10.00
Corn stalk	4.00	5.00	4.00	4.50	5.00	5.50
Sunflower seed skin	4.00	5.00	4.00	4.50	5.00	5.50
Alfalfa meal	10.00	11.00	13.00	13.50	12.00	12.00
Corn	27.00	26.50	32.00	31.00	38.00	39.00
Soybean meal	10.00	11.00	8.00	9.00	8.00	8.00
Germ meal	5.50	6.00	4.00	6.50	3.00	7.00
Cotton meal	6.00	6.00	5.50	6.50	3.00	4.00
Wheat bran	2.00	3.00				
Peanut cake			4.00	5.00	3.00	3.00
NaCl	0.50	0.50	0.50	0.50	0.50	0.50
NaHCO_3_	0.50	0.50	0.50	0.80	1.00	1.00
Limestone			0.50	0.70	0.50	0.50
CaHPO4	0.50	0.50	0.50	0.50	0.50	0.50
NH_4_Cl					0.50	0.50
4% premix^a^	3.00	3.00	3.00	3.00	3.00	3.00
Yeast culture	5.00	0.00	7.00	0.00	7.00	0.00
Total	100.00	100.00	100.00	100.00	100.00	100.00
Nutrient levels^b^						
DE/(MJ/kg)	10.48	10.45	10.83	10.78	11.12	11.08
DM	89.55	89.72	89.44	89.70	89.30	89.50
CP	14.81	14.91	15.27	16.13	13.41	13.38
NDF	32.64	32.89	30.00	29.73	28.86	28.22
ADF	21.78	22.25	19.73	19.73	18.70	18.17
Ca	1.36	1.37	1.42	1.51	1.32	1.30
P	0.44	0.45	0.42	0.44	0.39	0.44

a The premix provided the following per kg of diets: Fe 60 mg, Cu 12 mg, Zn 60 mg, Mn 45 mg, nicotinic acid 60 mg, I 0.6 mg, Se 0.2 mg, VA 3500 IU, VD 1200 IU, VE 20 IU, Ca 2 g, P 1 g, Co 20 mg, NaCl 5 g.

b DE was a calculated value, while other nutrient levels were measured values.

### Determination method of dietary nutrient levels

Dry matter (DM), crude protein (CP), neutral detergent fiber (NDF), acid detergent fiber (ADF), calcium (Ca) and phosphorus (P) in the diet were measured according to the Chinese national standards GOST 31640-2012, GB/T 6432-2018, GB/T 20806-2006, NY/T 1459-2007, GB/T 6436-2018 and GB/T 6437-2018, respectively. Digestible energy (DE) was calculated by Refs3000 software.

### Sample collection and detection

#### Determination of growth performance indexes

Feeding amounts and residual amounts of each sheep were accurately weighed every day, and the average daily feed intake (ADF) was calculated. All the 105 tested lambs were weighed before feeding in the mornings at 0, 30, 60, 90 and 130 days of the trial period. The average daily weight gain (ADG) and feed to weight gain ratio (F/G) was calculated (ADF / ADG), and the calculation formulas are provided below.

Average initial body weight (kg) = sum of initial weight of sheep in each group/number of sheep in each group;
Average final body weight (kg) = sum of final weights of sheep in each group /number of sheep in each group;
ADF (kg/day) = sum of feed intake of sheep in each group/number of days of the experiment;
ADG (g/day) = sum of the stage weight gain of sheep in each group/number of sheep in each group.

#### Serum sample collection

At the 0, 30, 60 and 90 days of the trial period, before feeding in the morning, six sheep from each group were selected for aseptic blood collection from the jugular vein with an aseptic needle and vacuum biochemical tube. The same six lambs per group were repeatedly sampled at the different time points. The sample tubes were set upright for 30 min to allow the blood to clot. The samples were then centrifuged at 3,000 r/min for 10 min. Subsequently, the upper serum was transferred to a 5 mL aseptic enzyme-free cryopreservation tube and stored in a −80°C refrigerator for the analysis of indexes of immune function, antioxidant capacity and hormone levels.

#### Analysis of serum indexes of immune function

Enzyme-linked immunosorbent assay (ELISA) kits were used to test the serum levels of immune indexes, including non-specific immune factors such as lysozyme (LZM) and cytokines interleukin-1β (IL-1β), IL-2, IL-4, IL-6, IL-10, interferon-γ (IFN-γ), tumor necrosis factor-α (TNF-α), as well as specific immune factors such as immunoglobulins IgG and IgM and soluble antigens (sCD3, sCD4, sCD8) of T lymphocyte subsets. The enzyme activities of acid phosphatase (ACP) were measured by spectrophotometry. All the above ELISA kits and enzyme activity spectrophotometry kits were purchased from Nanjing Jiancheng Bioengineering Research Institute, and the detection methods refer to the kit instructions. The UV spectrophotometer used in this test was the model T6 new century produced by Beijing Puxi General Instrument Co., Ltd. The microplate reader was the model 800TS-SN from Agilent Technologies, Ltd. (United States). The information of the kits is presented in Table 2.

**Table 2:** Serum immune index kits and their product numbers.

Items	Kit names	Product numbers
LZM	Lysozyme assay kit	A050-1-1
IL-1β	Interleukin -1β assay kit	H002-1-2
IL-2	Interleukin −2 assay kit	H003-1-2
IL-4	Interleukin −4 assay kit	H005-1-2
IL-6	Interleukin −6	H007-1-2
IL-10	Interleukin −10 assay kit	H009-1-2
IFN-γ	Interferon-γ assay kit	H025-1-2
TNF-α	Tumor Necrosis Factor-α assay kit	H052-1-2
IgG	Immunoglobulin G assay kit	H106-1-1
IgM	Immunoglobulin M assay kit	H109-1-2
sCD3	CD3 assay kit	H155
sCD4	CD4 assay kit	H156-1-1
sCD8	CD8 assay kit	H157-1-1
ACP	Acid phosphatase assay kit	A060-1-1

#### Analysis of serum indexes of antioxidant capacity

The serum antioxidant indexes were tested respectively, malondialdehyde (MDA) content by thiobarbituric acid method, superoxide dismutase (SOD) activity by xanthine oxidase method, catalase (CAT) activity by ammonium molybdate method, glutathione peroxidase (GSH-Px) activity by colorimetry, and the total antioxidant capacity (T-AOC) activity by Fe^3+^ reduction method. The information of the kits is presented in Table 3.

**Table 3:** Serum antioxidant index kits and their product numbers.

Items	Kit names	Product numbers
MDA	Malondialdehyde (MDA) assay kit (TBA method)	A003-1-2
SOD	Total Superoxide Dismutase (T-SOD) assay kit (Hydroxylamine method)	A001-1-2
CAT	CATalase (CAT) assay kit (Visible light)	A007-1-1
GSH-Px	Glutathione Peroxidase (GSH-Px) assay kit (Colorimetric method)	A005-1-2
T-AOC	Total antioxidant capacity assay kit	A015-1-2

#### Analysis of serum hormone levels

The serum levels of hormones were measured using ELISA kits, including glucocorticoid cortisol (Cort), growth hormone (GH), thyroid hormones (T3 and T4), insulin (INS) and leptin (LEP). The kits were purchased from Nanjing Jiancheng Bioengineering Institute. The information of the kits is presented in Table 4.

**Table 4:** Serum hormone kits and their product numbers.

Items	Kit names	Product numbers
Cort	Corticosterone assay kit	H205-1-2
GH	Growth hormone assay kit	H091-1-1
T3	Triiodothyronine assay kit	H222-1-2
T4	Thyroxine	H223-1-2
INS	INSulin assay kit	H203-1-2
LEP	LEPtin assay kit	H174-1-2

### Data analysis

The data were analysed as a completely randomized design with individual ram as the experimental unit. Meanwhile, the Durbin-Watson test was used to check the randomness of the initial and final body weight data to confirm the efficacy of the randomization process. The serum indicators of body weight (BW), average daily gain (ADG), average daily feed intake (ADF), feed to weight ratio (F/G), immune function, antioxidant capacity, and hormone levels were analyzed using the PROC MIXED program of SAS one-way ANOVA and t-test, with months as repeated measurement units and individual animals as experimental units. The model included the fixed effects of treatment, trial day/period and their interaction, and the random effect of ram. The data are presented as the least squares mean and pooled root mean square error (RMSE). A *p* < 0.05 was accepted as statistically significant.

## Results

### Effects on growth performance

It can be seen from Table 5 that there was no significant difference in ADF or F/G among all the three groups from Day 1 to 30 (*p* > 0.05). As for the ADG, there was no significant difference between the control group and YC group (*p* > 0.05), while that of BG group decreased by 8.05% (*p* < 0.05), compared with the control group. From Day 31 to 60, it still did not differ significantly in ADF or F/G among the three groups (*p* > 0.05), and the ADG of YC group and BG group increased by 8.02 and 4.76%, respectively, compared with the control group (*p* < 0.05). From Day 61 to 90, ADF of the three groups maintained no significant difference (*p* > 0.05), but the F/G in YC group were 5.87% higher (*p* < 0.05) and ADG 7.38% higher (*p* < 0.05) than those of the control group, respectively. However, as to the comparison between the BG group and control group, ADG or F/G did not display dramatic difference (*p* > 0.05). From Day 91 to 130, compared to the control group, the ADF and ADG of YC group ascended by 4.46 and 34.93% (*p* < 0.05), and those of BG group represented the same change trend by 9.41 and 32.10% (*p* < 0.05), while the F/G of these two treatment groups descended by 22.53 and 17.35% (*p* < 0.05), respectively. In general, over the whole 130-day test period, there was no significant difference in ADF among the three groups (*p* > 0.05). In addition, the ADG of YC group and BG group were 13.39% (39.96 g/d) and 6.73% (20.07 g/d) higher than that of the control group, respectively (*p* < 0.05), and the F/G of YC group was 10.33% lower than that of the control group (*p* < 0.05).

**Table 5:** Effects of YC and BG on growth performance of Mongolian ram lambs.

Items	Control	YC	BG	RMSE	*p-*value
Periods: Day 1–30					
Initial BW (kg)	12.738	12.704	12.718	1.093	0.9988
Final BW (kg)	20.416	20.526	19.778	1.104	0.5305
ADF (kg/d)	0.838	0.832	0.832	0.132	0.9966
ADG (g/d)	255.934^a^	260.732^a^	235.334^b^	10.374	0.0051
F/G ratio	3.262	3.182	3.522	0.420	0.4333
Periods: Day 30–60					
Initial BW (kg)	20.416	20.526	19.778	1.104	0.5305
Final BW (kg)	31.092	32.058	30.962	1.248	0.3487
ADF (kg/d)	1.442	1.448	1.438	0.185	0.9963
ADG (g/d)	355.866^b^	384.402^a^	372.798^a^	12.267	0.0104
F/G ratio	4.044	3.758	3.850	0.387	0.5100
Periods: Day 60–90					
Initial BW (kg)	31.092	32.058	30.962	1.248	0.3487
Final BW (kg)	41.772^b^	43.526^a^	41.236^b^	1.120	0.0180
ADF (kg/d)	1.942	1.962	1.908	0.116	0.7618
ADG (g/d)	356.000^b^	382.266^a^	342.534^b^	14.758	0.0035
F/G ratio	5.452^a^	5.132^b^	5.568^a^	0.158	0.0025
Periods: Day 90–130					
Initial BW (kg)	41.772^b^	43.526^a^	41.236^b^	1.120	0.0180
Final BW (kg)	51.524^c^	56.684^a^	54.120^b^	1.565	0.0008
ADF (kg/d)	2.022^c^	2.112^b^	2.208^a^	0.065	0.0026
ADG (g/d)	243.800^b^	328.950^a^	322.050^a^	17.220	<0.0001
F/G ratio	8.304^a^	6.430^c^	6.860^b^	0.204	<0.0001
Periods: Day 1–130					
Initial BW (kg)	12.738	12.704	12.718	1.093	0.9988
Final BW (kg)	51.524^c^	56.684^a^	54.120^b^	1.565	0.0008
ADF (kg/d)	1.560	1.588	1.598	0.575	0.9955
ADG (g/d)	298.354^c^	338.308^a^	318.478^b^	5.172	<0.0001
F/G ratio	5.228^a^	4.688^b^	5.008^ab^	0.320	0.0597

In the same row, values with no letter or the same letter superscripts mean no significant difference (*p* > 0.05), while different letter superscripts mean significant difference (*p* < 0.05). *n* = 35.

BW, body weight; ADG, average daily gain; ADF, average daily feed intake; F/G, ADF/ADG.

### Effects on serum indexes of immune function

#### Effects on serum indexes of non-specific immunity

To determine the effects of YC and BG on the non-specific immune function of Mongolian sheep, the serum LZM level and ACP activity, as well as the serum levels of some cytokines, were detected by ELISA and spectrophotometry.

**Figure 1:**
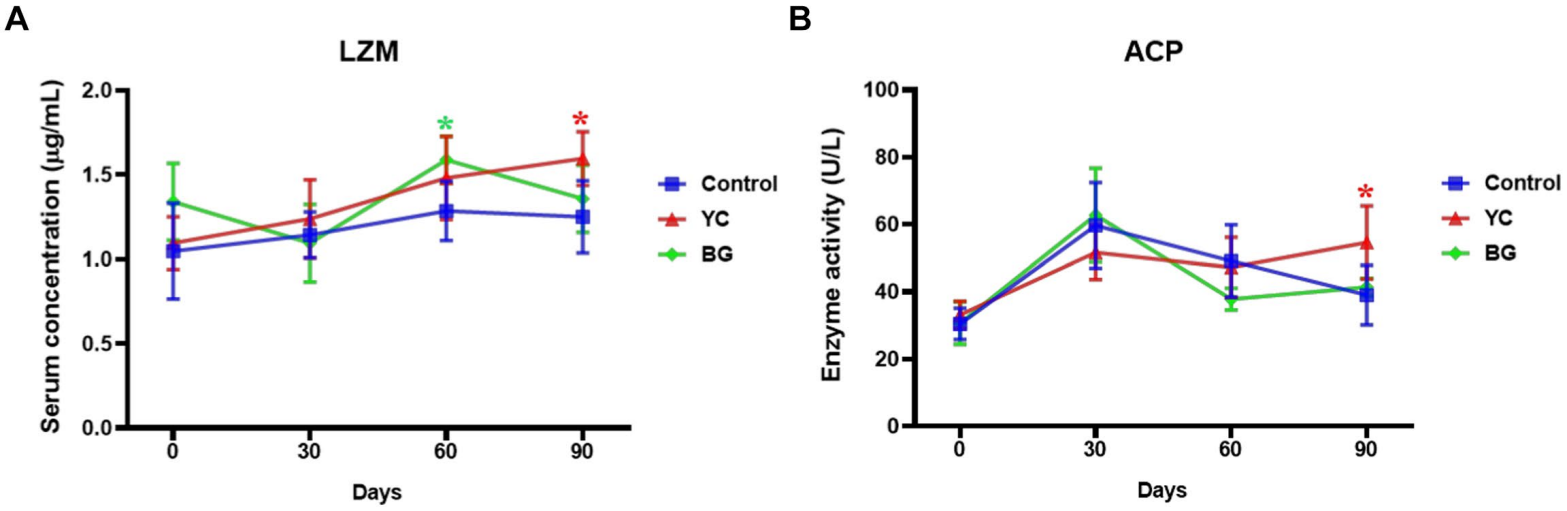
Effects of YC and BG on the serum LZM level and ACP activity in Mongolian ram lambs. **(A)** Effects on the serum LZM concentration. **(B)** Effects on the serum ACP enzyme activity. Mean ± SEM; * (*p* < 0.05) vs. Control; ** (*p* < 0.01) vs. Control. T-test. *n* = 6.

As shown in Figure 1, the serum LZM concentration was significantly promoted by YC feeding on the 90th day (*p* < 0.05), while thus similar facilitating effect of BG feeding was observed on the 60th day (*p* < 0.05). The serum ACP enzyme activity in YC group was slightly higher than that in the control group on the 90th day, and yet there was no significant difference in the serum ACP activities between these two experimental groups and the control group at the other time points (*p* > 0.05). In summary, both serum indexes of non-specific immunity were enhanced by YC feeding on the 90th day.

**Figure 2:**
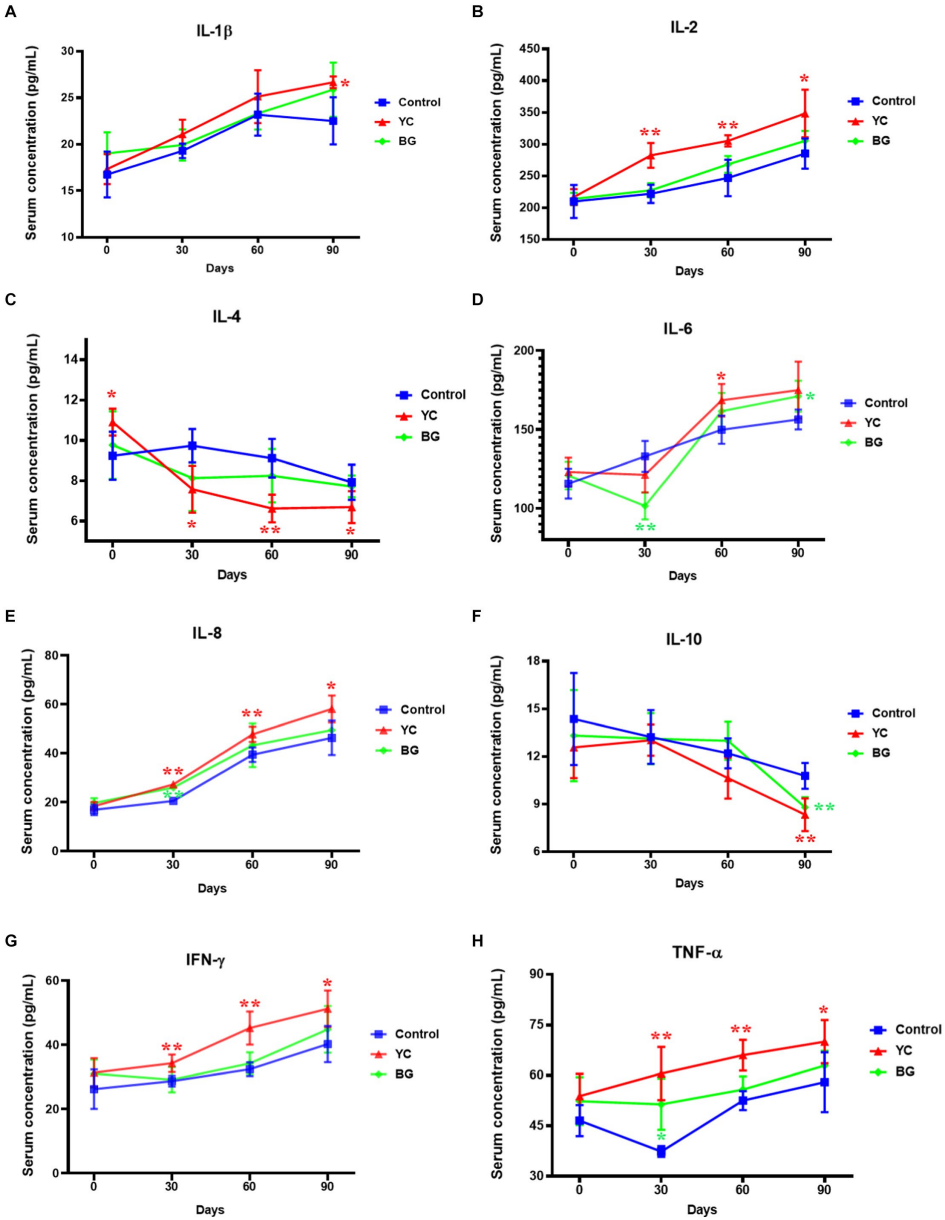
Effects of YC and BG on the serum levels of cytokines in Mongolian ram lambs. **(A)** Effects on the serum IL-1β concentration. **(B)** Effects on the serum IL-2 concentration. **(C)** Effects on the serum IL-4 concentration. **(D)** Effects on the serum IL-6 concentration. **(E)** Effects on the serum IL-8 concentration. **(F)** Effects on the serum IL-10 concentration. **(G)** Effects on the serum IFN-γ concentration. **(H)** Effects on the serum TNF-α concentration. Mean ± SEM; * (*p* < 0.05) vs. Control; ** (*p* < 0.01) vs. Control. T-test. *n* = 6.

As to the serum levels of cytokines (Figure 2), the IL-1β content in sheep serum of YC group was just marginally higher than that of the control group on Day 90 (*p* < 0.05). Moreover, the IL-2 was also sensitive to YC addition, with the serum levels of the former being substantially increased by the latter on the 30th, 60th and 90th days (*p* < 0.05), to different extents. Additionally, the serum contents of IL-8, IFN-γ and TNF-α showed similar ascend trends to those of IL-2 at the corresponding time points (*p* < 0.05). By contrary, the IL-4 levels were slightly decreased by YC feeding on Day 30 to 90 (*p* < 0.05). Interestingly, the influences of these two feed additives on IL-6 concentration were indefinite, as serum IL-6 was downregulated on the 30th day but upregulated on the 90th day in BG group, while being improved on the 90th day in YC group. Compared with the control group, IL-10 levels in the serum of both YC and BG groups were markedly restrained only on the 90th day (*p* < 0.05). Generally speaking, serum concentrations of the tested cytokines were more sensitive to YC feeding than BG addition, and the effects on most of the cytokines lasted from Day 30 to Day 90, such as increased IL-2, IL-8, IFN-γ and TNF-α, as well as decreased IL-4. In addition, the influences of YC feeding on several cytokines only occurred on Day 90, such as promoted IL-1β and repressed IL-10.

#### Effects on serum indexes of specific immunity

In this study, we explored the effects of YC and BG on the specific immunity of Mongolian sheep by measuring the serum concentrations of immunoglobulins (IgG and IgM) for the capacity of humoral immunity and soluble T lymphocyte antigens (sCD3, sCD4 and sCD8) for that of cellular immunity.

**Figure 3:**
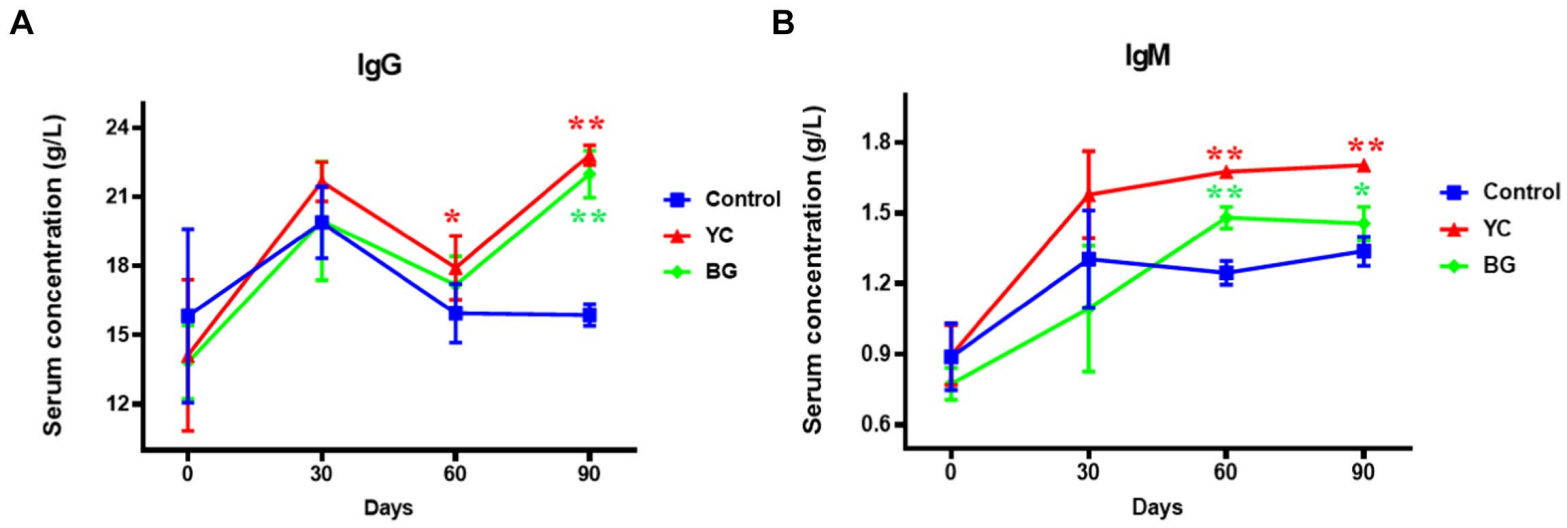
Effects of YC and BG on the serum levels of IgG and IgM in Mongolian ram lambs. **(A)** Effects on the serum IgG concentration. **(B)** Effects on the serum IgM concentration. Mean ± SEM; * (*p* < 0.05) vs. Control; ** (*p* < 0.01) vs. Control. T-test. *n* = 6.

As illustrated in Figure 3, compared to the control group, the serum IgG level in YC group was marginally enhanced on Day 60 (1.12 times) and kept this increasing action but in even more dramatic degree on Day 90 (1.44 times). Moreover, the serum IgG concentration in BG group also showed a similar upward trend on Day 90 (*p* < 0.05). The serum IgM content of both YC and BG groups was remarkably higher than that of the control group on the 60th and 90th days, respectively, with more increase in YC group than in BG group at both time points. Altogether, the serum levels of IgG and IgM were found to be upregulated by both YC and BG feeding in varying degrees on the 90th day.

**Figure 4:**
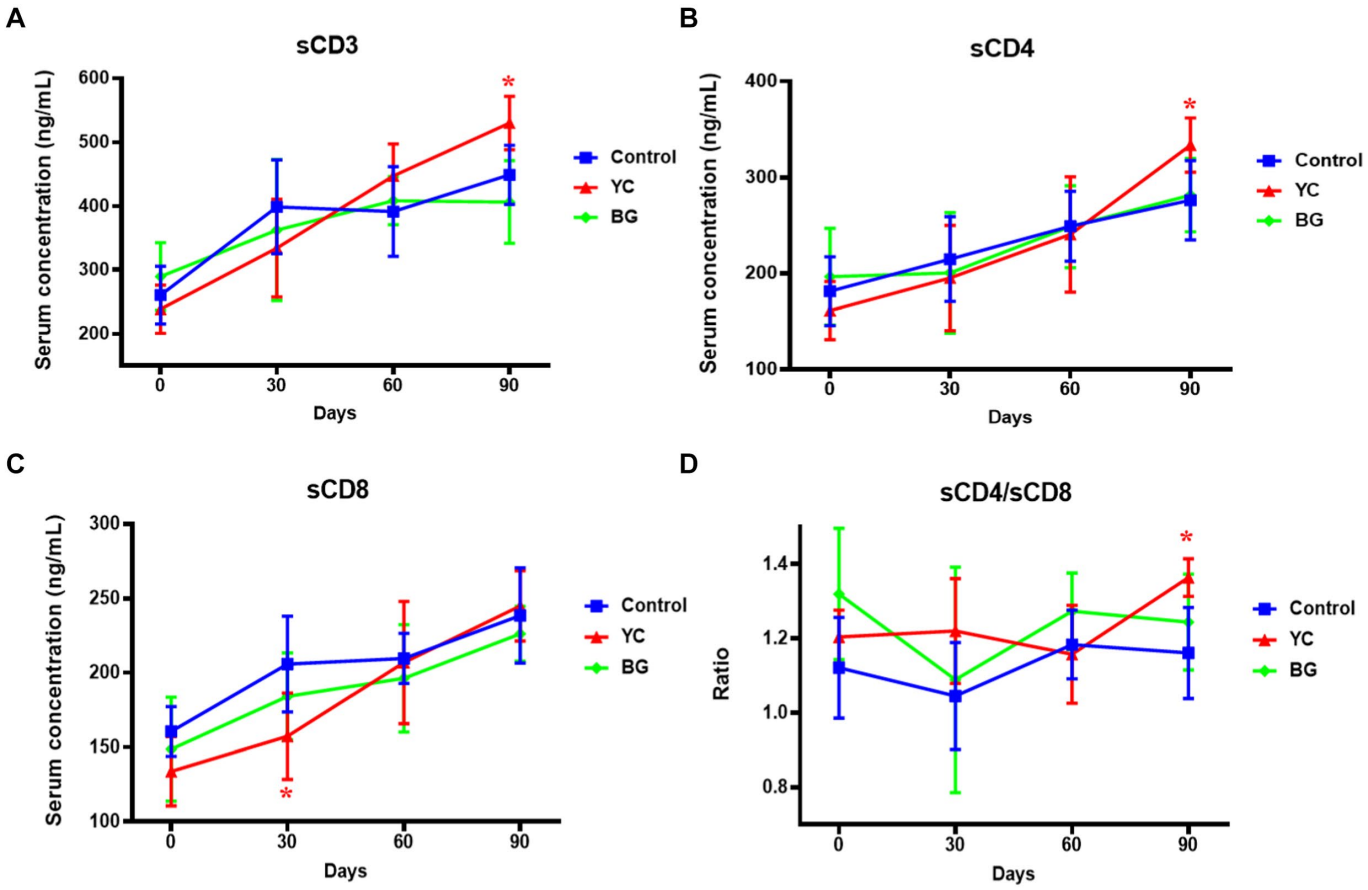
Effects of YC and BG on the serum levels of soluble T lymphocyte antigens in Mongolian ram lambs. **(A)** Effects on the serum concentration of sCD3. **(B)** Effects on the serum concentration of sCD4. **(C)** Effects on the serum concentration of sCD8. **(D)** Effects on the ratio of sCD4/sCD8. Mean ± SEM; * *p* < 0.05) vs. Control; ** (*p* < 0.01) vs. Control. T-test. *n* = 6.

Figure 4 illustrated the effects of YC and BG on soluble T lymphocyte antigens in Mongolian sheep. The results showed that the sCD8 level in YC group was significantly lower than that of the control group on Day 30 (*p* < 0.05), while the serum concentrations of sCD3, sCD4, and the ratio of sCD4/sCD8, of YC group were rather higher than those of control group on Day 90 (*p* < 0.05). Furthermore, there was no significant difference in serum contents of sCD3, sCD4 and sCD, or the comparative value of sCD4/sCD8, between the control group and YC group or BG group on Day 60 (*p* > 0.05). In short, YC addition in feed of mutton sheep may regulate serum soluble T lymphocyte antigens to affect the specific immune function, mainly by elevating the serum levels of sCD3 and sCD4.

### Effects on serum indexes of antioxidant capacity

**Figure 5:**
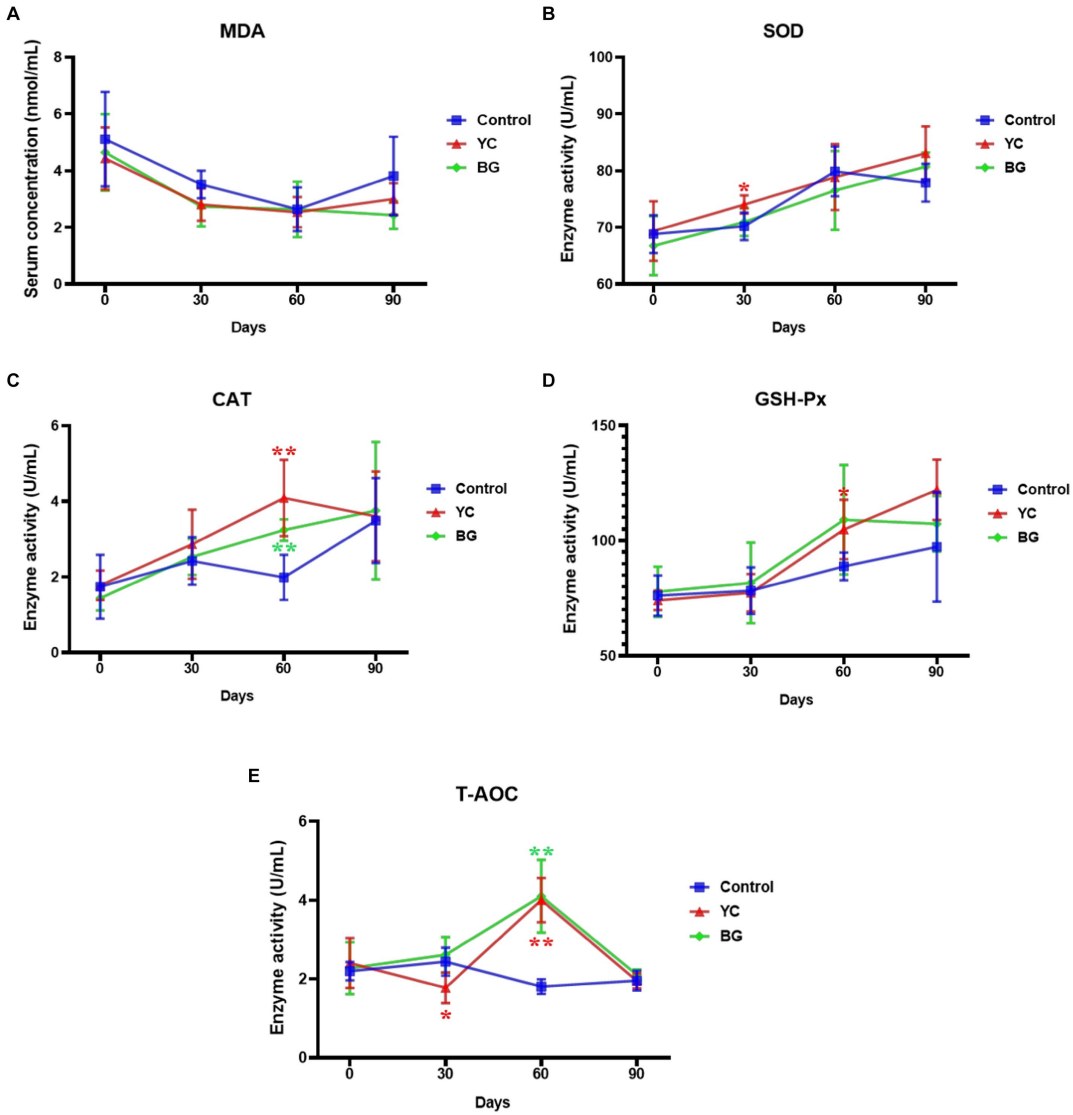
Effects of YC and BG on the serum indexes of antioxidative status in Mongolian ram lambs. **(A)** Effects on the serum MDA concentration. **(B)** Effects on the serum SOD activity. **(C)** Effects on the serum CAT activity. **(D)** Effects on the serum GSH-Px activity. **(E)** Effects on the serum T-AOC activity. Mean ± SEM; * (*p* < 0.05) vs. Control; ** (*p* < 0.01) vs. Control. T-test. *n* = 6.

To estimate the effects of YC and BG additives on the antioxidative function of mutton ram lambs, the enzyme activities of SOD, CAT, GSH-Px and T-AOC in sheep serum, as well as serum MDA content, were determined through a variety of methods. The results displayed in Figure 5 showed that there was no significant difference in serum MDA content between the control group and any experimental group at each time point (*p* > 0.05). To be noted, the SOD activity in the serum of YC group was significantly higher than that of the control group on Day 30. Compared to the control group, both YC and BG feedings could notably increase serum CAT activity on Day 60, while GSH-Px activity was just slightly promoted by YC addition at the same time point (*p* < 0.05). Furthermore, the activity of T-AOC was slightly reduced in YC group on Day 30, but remarkably induced in both test groups on Day 60, compared with the control group. In brief, the serum enzyme activities of all of the tested antioxidative indexes, SOD, CAT, GSH-Px and T-AOC, were raised in varying degrees on Days 30 or 60 by YC addition in sheep feed, while CAT and T-AOC activities were markedly enhanced on Day 60 by BG addition.

### Effects on serum hormone levels

**Figure 6:**
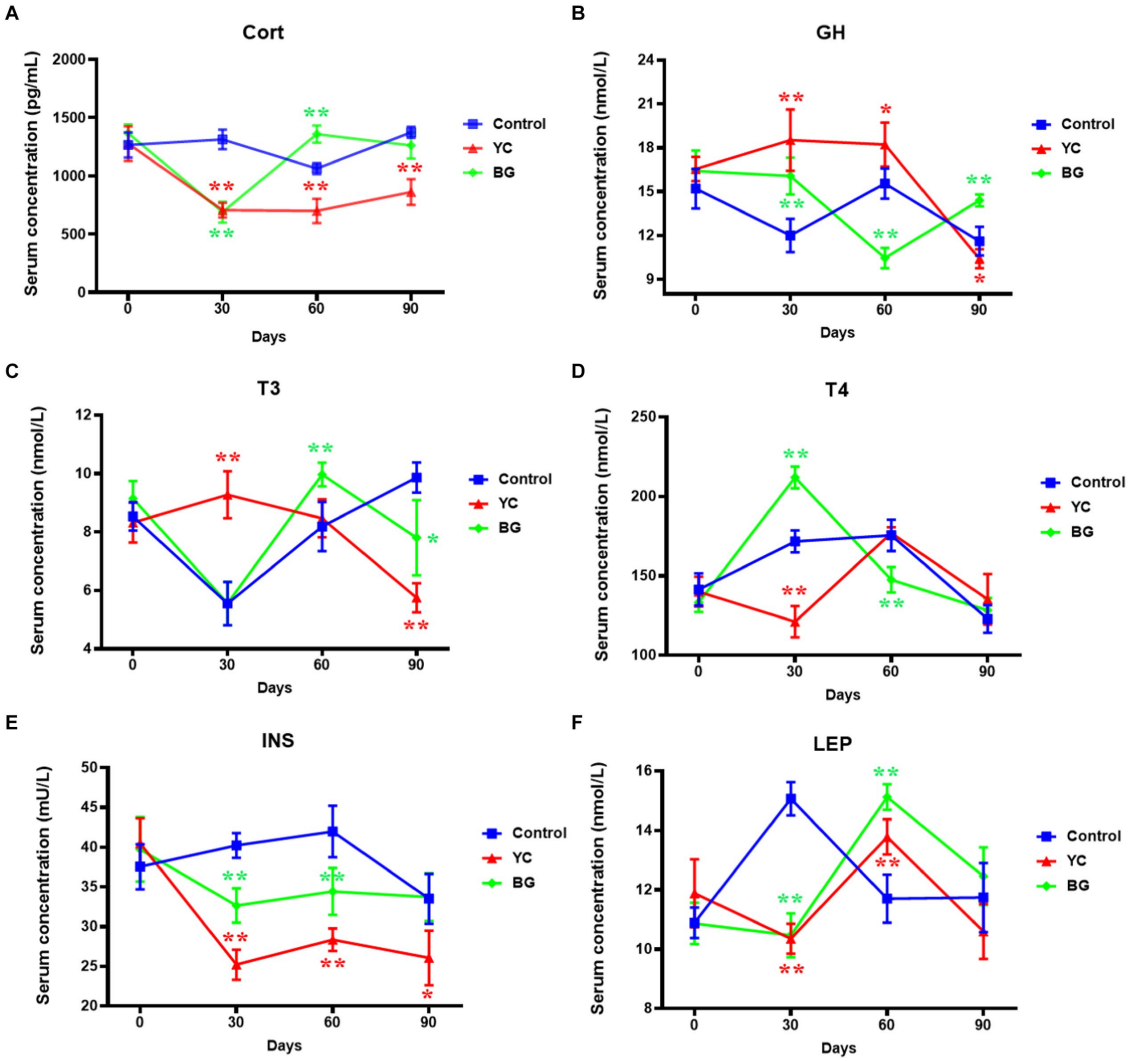
Effects of YC and BG on the serum hormone levels in Mongolian ram lambs. **(A)** Effects on the serum Cort concentration. **(B)** Effects on the serum GH concentration. **(C)** Effects on the serum T3 concentration. **(D)** Effects on the serum T4 concentration. **(E)** Effects on the serum INS concentration. **(F)** Effects on the serum LEP concentration. Mean ± SEM; * (*p* < 0.05) vs. Control; ** (*p* < 0.01) vs. Control. T-test. *n* = 6.

To further and more widely study the effects of YC and BG added in the feed of ram lambs, the metabolism-associated hormone levels of Cort, GH, T3, T4, INS and LEP in the serum of Mongolian sheep were admeasured by ELISA method. As shown in Figure 6, the serum Cort levels in YC group were significantly lower than those in the control group at all of the tested time points, and those of BG group showed a same trend with a similar extent to YC group on Day 30 but a opposite result on Day 60. Compared with the control group, the average serum concentration of GH in YC group markedly increased on Days 30 and 60 but slightly decreased on Day 90, while that in BG group presented a varied and complicated trend, up on Days 30 and 90 but down on Day 60. The change trends of serum T3 levels in the two experimental groups resembled each other, manifested as ascended in earlier stage (YC group on Day 30 and BG group on Day 60) and then descended in later stage (both on Day 90). Compared to the control group, the serum T4 concentration in YC group was notably lower on Day 30, and that in BG group were evidently higher on Day 30 but slightly lower on Day 60. In addition, serum INS contents were significantly reduced by YC feeding in various degrees at different time points, and those were also repressed by BG feeding to a lesser extent than in YC group on the 30th and 60th days. Moreover, LEP in sheep serum of both YC and BG groups were remarkably downregulated on Day 30 but obviously upregulated on Day 60. In summary, the serum levels of the tested metabolism-associated hormones were significantly affected by YC and BG additives, some showing short-term effects, such as T4 and LEP, while the others presenting long-term actions, such as Cort, GH, T3 and INS. Furthermore, the sheep serum hormones were more sensitive to YC than BG addition in feed, and as to the administration time points, the effectiveness was most remarkable on Day 30, displaying increased (GH and T3) or decreased (Cort, T4, INS and LEP) trends.

## Discussion

In the study presented here, we assessed the impacts of YC and one of its active constituents, β-glucan, as feed supplements on a range of parameters associated with growth and health, including growth performance, immune function, antioxidant capacity and hormonal levels, in the Mongolian mutton ram lambs. On this basis, it was proved that the feed additive YC can be used as a suitable alternative to antibiotics in ruminant feeding.

### Effects on growth performance

It has been proved that YC is a useful feed additive for manipulation of rumen fermentation in lambs fed with high-concentrate diets, with enhanced crude protein and cell wall digestibility, ruminal molar proportion of propionate and plasma glucose concentration (17). One study showed that YC supplementation resulted in 31.1 g/d more ADG than the control group with unchanged feed intake during a 56-day test period in fattening lambs, which could be attributed to improved digestibility of neutral detergent fiber and acid detergent fiber (18). In the present study, ADF changes during the entire 130-day test period were neither caused by the addition of YC nor by the addition of BG, while both of them could obviously increase the ADG of Mongolian sheep, with 39.96 g/d more ADG in YC group and 20.07 g/d more ADG in BG group, which is in line with previous studies. On the other hand, fed with YC could remarkably decrease the F/G ratio of ram lambs. Our previous results *in vitro* showed that YC can increase the gas production of rumen fluid and the concentrations of total VFA, acetic acid, propionic acid, butyric acid, ammonia nitrogen, bacterial protein and other nutrients, and it could enhance the digestibility of dry matter, crude protein, neutral detergent fiber and acid detergent fiber of the diet (19). These results indicated that YC can promote rumen fermentation function and improve the *in vitro* digestibility of dietary nutrients, which should be one of the reasons for improving the growth performance of Mongolian mutton ram lambs.

### Effects on serum indexes of immune function

LZM and ACP are crucial non-specific immune factors, both of which play an active role in maintaining non-specific immune balance and resisting pathogens of the animal body (20). The results of the present study showed that the LZM content and ACP activity in the serum of mutton ram lambs fed with YC were remarkedly higher than those of the control group, suggesting that YC may stimulate the LZM production and simultaneously enhance the ACP activity, thereby improving the antibacterial defense function and promoting the disease resistance of the body.

Although the contents of cytokines in animal serum are low, they play an important role in the process of body defense against diseases. As an inflammatory factor, IL-1β can promote the growth and differentiation of B lymphocytes, being conducive to the formation of antibodies and improving the anti-infection effects of the body (21). IL-2 can activate T lymphocytes, increase the cytotoxicity of natural killer cells, and promote cytokine production, thus playing an important role in the immune response and anti-virus process of the body (22). TNF-α is synthesized and secreted by monocytes and macrophages, which can promote the proliferation of B cells and enhance the immune response efficiency of T cells, thereby killing tumor cells or inhibiting their proliferation (21). Proinflammatory cytokines, such as IL6 and IFN-γ, play a pivotal part in the inflammatory process, as IL-6 promoting the proliferation and differentiation of B lymphocytes to produce antibodies and IFN-γ exerting antiviral functions and enhancing the phagocytosis of macrophages (23). Therefore, an appropriate increase in the contents of inflammatory mediators is conducive to promoting the body’s immune response. We found that the serum contents of the cytokines such as IL-1β, IL-2, IL-6, IL-8, IFN-γ and TNF-α were obviously increased by YC addition at different time points while those of IL-4 and IL-10 were evidently decreased. Furthermore, our findings illustrated that compared to the control group, the levels of IL-8 and TNF-α in the BG group were clearly higher while that of IL-6 were substantially lower on Day 30. One potential explanation for this phenomenon may lie in that some ingredients in the YC such as β-glucan and mannan bind to surface mode receptors in cells including macrophages and neutrophils, which activated macrophages and neutrophils and regulated the expression of antioxidant and inflammatory factors (24, 25).

Serum immunoglobulin is produced by B lymphocytes, which is with the ability to enhance anti-virus and prevent infection and of importance in the immune response process (26). In this work, the results demonstrated that adding YC obviously elevated the serum contents of IgG and IgM in ram lambs on Day 90, indicating that YC can improve the specific immune function of the body. It was previously reported that supplementation with *Saccharomyces cerevisiae* products increased serum concentrations of total protein, IgA and IgG (27), which is in line with our data, suggesting that yeast fermented products such as YC could help ruminants to promote immunity and resistance to pathogens. Ma et al. found that adding β-glucan to calf diet can increase the serum concentrations of immunoglobulins and stimulate alkaline phosphatase, thereby enhancing immunity in dairy calves (28). Similarly, our results showed that β-glucan supplement markedly heightened the serum levels of IgG and IgM in Mongolian mutton ram lambs, which may be due to the fact that β-glucan can improve the disease resistance of animals by adsorbing toxins and pathogenic microorganisms (29).

A mass of data have illustrated that it is an important way to detect the T lymphocyte subsets for evaluating the cellular immune level of animals, and its stability is a crucial factor to maintain the specific immune regulation of the body (30). Among all the subsets, CD_3_^+^ lymphocytes represent the total number of T cells in peripheral blood circulation and reflect the function of cellular immunity. Further, the ratio of CD_4_^+^/CD_8_^+^ T cells is a vital index to estimate the strength of cellular immune function. The levels of sCD3, sCD4 and sCD8 in serum have been considered as indicators of T cell subset activation and may be important in monitoring and characterizing disease processes during immunological diseases (31). In this research, YC supplemented in the diet elevated the serum concentrations of sCD3 and sCD4 on Day 90, as well as the ratio of sCD4 and sCD8, indicating that YC could regulate the levels of T lymphocyte subset activation to affect the specific immunity of the Mongolian ram lambs. In addition, BG feeding displayed no significant influence on the serum concentrations of T lymphocyte antigens. These results indicate that YC administration in the feed could promote the activation and function of T lymphocytes, thereby improving the specific immune function of mutton sheep; nevertheless, such effect may be not attributed of BG.

### Effects on serum indexes of antioxidant capacity

MDA is a biomarker of lipid peroxidation, and its content directly reflects the degree of lipid peroxidation and the metabolism of free radicals in the body and indirectly reflects the extent of oxidative damage to cells and tissues (32). On the other hand, the animal body is equipped with an enzymatic system for scavenging free radicals, including SOD, CAT, GSH-Px and other antioxidant enzymes, which serves to maintain the dynamic balance of redox (33). SOD can eliminate superoxide anion free radicals and protect cell membranes from damage; CAT can remove hydrogen peroxide (H_2_O_2_) in the body, so as to protect cells from the toxicity of H_2_O_2_; GSH-Px can protect macromolecular components of tissues from oxygen free radicals by removing reactive oxygen intermediates. In previous reports, the effects of YC supplementation in the diet on the MDA or T-AOC in serum of ruminants were inconsistent, with reduced or unchanged MDA contents, as well as increased or decreased T-AOC levels (34, 35). In this study, our data illustrated that the MDA content in the serum of sheep in YC group was not significantly affected throughout the whole trial period. Moreover, it was discovered that the antioxidative enzyme activities in the serum, including CAT, GSH-Px and SOD, were markedly augmented by YC added in sheep feed, and the T-AOC level was dramatically higher than that of the control group on Day 60. No differences were observed between the control and two test groups in plasma levels of MDA during the whole test period, suggesting that the induction of the antioxidant enzymes and the increase of T-AOC were effectively stimulated by oxidative stress, rather than an unchanged antioxidant status (36). The antioxidant effect of YC may be due to its rich variety of nutrients, including vitamins, microelements, enzymes, peptides, and polysaccharides (β-glucan and mannan). For example, Wang et al. used *Escherichia coli* O141: K99 to induce oxidative stress in 28-day-old preweaning calves, and discovered that β-glucan might effectively improve the antioxidant function of the calves and reduce the incidence of oxidative stress after *Escherichia coli* O141: K99 infection (37). We found that the serum MDA concentrations in BG group did not alter compared to that in the control group, while the CAT activity and T-AOC levels were upregulated by BG administration.

### Effects on serum hormone levels

Evaluation of the hormone profile may provide considerable clues for the influence of YC and β-glucan administrations on metabolic processes. In this study, we measured the levels of several metabolism-related hormones, including Cort, GH, T3, T4, INS, and LEP. Cort belongs to the glucocorticoid family of adrenal cortical hormones, and its level is often used as an indicator to measure the degree of stress and changes in physiological functions of animals (38). GH is an important hormone to promote animal growth, which can accelerate the synthesis and deposition of protein and inhibit its degradation, thereby increasing the volume and number of cells and promoting the growth of the body (39). The two main thyroid hormones, T3 and T4, can stimulate the production of protein, as well as the absorption of glucose and the decomposition of lipid in the intestinal mucosa, thus promoting the growth and development of animal body, and the serum T3 level is positively correlated with the growth of organisms (40). INS is a crucial hormone to improve the anabolism of glycogen, fat and protein and to maintain the relative stability of blood glucose, thus playing a notable part in animal growth (41). In addition to regulating body weight, the abilities of LEP are more far-reaching and include profound glucose-lowering and anti-lipogenic effects (42).

The data from our research represented that the effects of YC and BG additives on the serum hormone levels were rather complicated. The serum Cort levels were significantly reduced by fed with YC during the whole experimental period. Conversely, compared to the control group, the Cort content in BG group showed a substantial downward trend on Day 30 but rising in a small degree on Day 60. The addition of YC to the diet appears to inhibit the secretion of cortisol, indicating that YC may reduce the stress response of ram lambs, reduce the occurrence of diseases, and further promote the growth and development of the body. Both YC and BG administration remarkably increased GH concentrations in the sheep serum on Day 30, indicating that both feed additives could effectively promote the growth and development of sheep and improve their growth performance. The serum T3 levels apparently ascended on Day 30 in YC group and on Day 60 in BG group, as by contrary, the serum T4 concentrations apparently descend at such time points. The observed increases in GH and T3 levels in Mongolian ram lambs fed with YC or BG revealed the stimulatory effect of thus two feed additives on anabolism. Furthermore, it is generally believed that T4 plays its biological role by transforming it into T3, yet whether the decline in T4 levels is related to this mechanism remains to be further studied. The serum INS concentrations in the YC and BG groups showed some certain degrees of reduction on Days 30 and 60, indicating that both additives may regulate the serum INS levels to promote glucose absorption and decrease glucose output along with increasing glycogen synthesis. The serum LEP contents in sheep of YC and BG groups were obviously lower on Day 30 but clearly higher on Day 60 than that of the control group, suggesting that YC and BG administration could increase serum glucose levels and promote adipogenesis in the earlier period, but presented some feedback regulation in the later period. Taken all the results together, it was speculated that YC additive may potentially enhance serum levels of hormones related to anabolic pathways such as GH and thyroid hormones, repress hormones related to catabolic pathways such as INS and LEP, and reduce the stress response through inhibiting the secretion of Cort, which may indirectly promote the growth and development of Mongolian sheep.

The present study investigated the effects of YC on the growth, immunity, antioxidant capacity and hormone levels of Mongolian ram lambs from the macro level. However, there has been a lack of in-depth study of the corresponding mechanisms, which will be the focus of the follow-up study of our research group.

## Conclusion

The effects of yeast culture and one of its main active ingredients, β-glucan, on growth performance, immune function, antioxidant capacity and hormone levels of Mongolian ram lambs were evaluated *in vivo* in the present study. The results showed that yeast culture and β-glucan added to the sheep diet could improve the growth performance, immune function and antioxidant capacity of Mongolian sheep and regulate the serum levels of metabolism-associated hormones. Therefore, YC could be considered as a potential substitute for dietary subtherapeutic antibiotics and can be used as an animal feed additive. These data provided theoretical support for the clinical application of this preparation in sheep husbandry.

## Data availability statement

The data presented in the study are deposited in the figshare dryad digital repository, accession number https://doi.org/10.6084/m9.figshare.26196551.

## Ethics statement

The animal study was approved by all animal experiments were carried out in compliance with the experimental practices and standards approved by the animal welfare and research ethics committee of Inner Mongolia Agricultural University (Approval ID:2022104 and 2020009). The study was conducted in accordance with the local legislation and institutional requirements.

## Author contributions

HC: Writing – original draft, Visualization, Validation, Methodology, Investigation, Formal analysis, Data curation, Conceptualization. SxL: Writing – original draft, Validation, Resources, Methodology. SoL: Conceptualization, Data curation, Writing – original draft, Writing – review & editing. DoL: Data curation, Writing – original draft. XL: Software, Writing – review & editing. ZX: Investigation, Methodology, Writing – original draft, Writing – review & editing. DaL: Supervision, Writing – original draft, Visualization, Validation, Methodology, Investigation, Formal analysis, Data curation, Conceptualization.
