# Supplementary material for: Effects of yeast culture on growth performance, immune function, antioxidant capacity and hormonal profile in Mongolian ram lambs
Source: Front Vet Sci. 2024 Jul 23;11:1424073. doi: 10.3389/fvets.2024.1424073 (PMC11301782; doi:10.3389/fvets.2024.1424073)
Supplement: Supplementary file 1 [file Table_1.docx]

**Effects of Yeast Culture on Growth Performance, Immune Function, Antioxidant Capacity and Hormonal Profile in Mongolian** **Ram Lambs**

Hui. Chen, Shixiong Liu, Songjian Li, Dongfang Li, Xueqiang Li, Zixuan Xu, Dacheng Liu

*animal* journal

**Supplementary material**

**Supplementary Material S1. Mathematical equations**

The calculation formulas are as follows:

Average initial weight (kg) = total initial weight of each sheep/number of sheep in captivity;

Average final weight (kg) = total final weight of each sheep/number of sheep in captivity;

Average daily weight gain (g/d) = total weight gain in each stage/(number of days in each stage × Number of sheep in the captivity);

Average daily feed intake (kg/d) = total feed intake/total trial days;

Feed to weight ratio = average daily feed intake/average daily gain.

**Supplementary Material S2. Codes of statistical models**

The SAS codes used are as follows:

**130d ram weight**

data ss;

do rep=1 to 5;

do treat=1 to 3;

input y@@;

output;

end;

end;

cards;

58.6 55.96 50.13

55.01 54.56 52.76

58.43 51.89 52.74

54.84 53.13 50.49

56.54 55.06 51.5

;

proc anova;

class treat rep;

model y=treat;

means treat/duncan;

means treat;

Run;

**90d ram weight**

data ss;

do rep=1 to 5;

do treat=1 to 3;

input y@@;

output;

end;

end;

cards;

44.97 42.77 42.09

43.94 42.24 41.9

42.06 40.31 43.03

42.67 39.9 41.4

43.99 40.96 40.44

;

proc anova;

class treat rep;

model y=treat;

means treat/duncan;

means treat;

Run;

**60d ram weight**

data ss;

do rep=1 to 5;

do treat=1 to 3;

input y@@;

output;

end;

end;

cards;

33.87 31.52 32.91

32.01 31.71 30.9

30.23 29.71 30.06

31.72 29.41 31.02

32.46 32.46 30.57

;

proc anova;

class treat rep;

model y=treat;

means treat/duncan;

means treat;

Run;

**30d ram weight**

data ss;

do rep=1 to 5;

do treat=1 to 3;

input y@@;

output;

end;

end;

cards;

20.97 20.97 20.64

18.83 20.78 21.99

19.94 18.81 19.28

21.73 18.51 20.51

21.16 19.82 19.66

;

proc anova;

class treat rep;

model y=treat;

means treat/duncan;

means treat;

Run;

**0d ram weight**

data ss;

do rep=1 to 5;

do treat=1 to 3;

input y@@;

output;

end;

end;

cards;

13.57 14.16 14

11.34 13.74 12.89

13.47 12.21 11.43

12.01 11.14 13.44

13.13 12.34 11.93

;

proc anova;

class treat rep;

model y=treat;

means treat/duncan;

means treat;

Run;

**30d feed intake of rams**

data ss;

do rep=1 to 5;

do treat=1 to 3;

input y@@;

output;

end;

end;

cards;

0.88 0.89 0.88

0.88 0.88 0.88

0.97 0.95 0.97

0.83 0.82 0.84

0.6 0.62 0.62

;

proc anova;

class treat rep;

model y=treat;

means treat/duncan;

means treat;

Run;

**60d feed intake of rams**

data ss;

do rep=1 to 5;

do treat=1 to 3;

input y@@;

output;

end;

end;

cards;

1.24 1.23 1.24

1.29 1.32 1.32

1.47 1.43 1.43

1.55 1.5 1.50

1.69 1.71 1.72

;

proc anova;

class treat rep;

model y=treat;

means treat/duncan;

means treat;

Run;

**90d feed intake of rams**

data ss;

do rep=1 to 5;

do treat=1 to 3;

input y@@;

output;

end;

end;

cards;

1.82 1.73 1.76

1.98 1.89 1.91

1.94 1.88 1.92

2.02 1.98 2.01

2.05 2.06 2.11

;

proc anova;

class treat rep;

model y=treat;

means treat/duncan;

means treat;

Run;

**130d feed intake of rams**

data ss;

do rep=1 to 5;

do treat=1 to 3;

input y@@;

output;

end;

end;

cards;

2.06 2.13 2.0

2.04 2.18 2.0

2.15 2.18 2.11

2.21 2.26 2.05

2.10 2.29 1.95

;

proc anova;

class treat rep;

model y=treat;

means treat/duncan;

means treat;

Run;

**1-130d feed intake of rams**

data ss;

do rep=1 to 4;

do treat=1 to 3;

input y@@;

output;

end;

end;

cards;

0.83 0.83 0.84

1.45 1.44 1.44

1.96 1.91 1.94

2.11 2.21 2.02

;

proc anova;

class treat rep;

model y=treat;

means treat/duncan;

means treat;

Run;

**30d Ram Daily Growth**

data ss;

do rep=1 to 5;

do treat=1 to 3;

input y@@;

output;

end;

end;

cards;

272 227 266.33

256.33 234.67 240

261.33 249.33 254

264.33 220 257.67

249.67 245.67 261.67

;

proc anova;

class treat rep;

model y=treat;

means treat/duncan;

means treat;

Run;

**60d Ram Daily Growth**

data ss;

do rep=1 to 5;

do treat=1 to 3;

input y@@;

output;

end;

end;

cards;

404.67 383 364

376.67 364.33 346

368 390 346.33

392.67 363.33 363.67

380 363.33 359.33

;

proc anova;

class treat rep;

model y=treat;

means treat/duncan;

means treat;

Run;

**90d Ram Daily Growth**

data ss;

do rep=1 to 5;

do treat=1 to 3;

input y@@;

output;

end;

end;

cards;

370 343.67 337.33

384.33 351 369

397.67 314.67 366.67

365 353.33 361

394.33 350 346

;

proc anova;

class treat rep;

model y=treat;

means treat/duncan;

means treat;

Run;

**130d Ram Daily Growth**

data ss;

do rep=1 to 5;

do treat=1 to 3;

input y@@;

output;

end;

end;

cards;

340.75 329.75 243.25

361 320.5 266.25

315 340 240

308.5 320.5 227.25

319.5 299.5 242.25

;

proc anova;

class treat rep;

model y=treat;

means treat/duncan;

means treat;

Run;

**1-130d Ram Daily Growth**

data ss;

do rep=1 to 5;

do treat=1 to 3;

input y@@;

output;

end;

end;

cards;

346.38 321.54 298.15

345.85 317.85 302.31

333.92 324.77 297

330.77 314.77 296.62

334.62 313.46 297.69

;

proc anova;

class treat rep;

model y=treat;

means treat/duncan;

means treat;

Run;

**30d ram feed weight ratio**

data ss;

do rep=1 to 5;

do treat=1 to 3;

input y@@;

output;

end;

end;

cards;

3.57 3.81 3.64

3.33 3.62 3.36

3.37 3.75 3.42

3.24 3.61 3.31

2.40 2.82 2.58

;

proc anova;

class treat rep;

model y=treat;

means treat/duncan;

means treat;

Run;

**60d ram feed weight ratio**

data ss;

do rep=1 to 5;

do treat=1 to 3;

input y@@;

output;

end;

end;

cards;

4.18 4.38 4.73

3.95 3.92 4.12

3.87 3.93 3.98

3.42 3.63 3.81

3.37 3.39 3.58

;

proc anova;

class treat rep;

model y=treat;

means treat/duncan;

means treat;

Run;

90**d ram feed weight ratio**

data ss;

do rep=1 to 5;

do treat=1 to 3;

input y@@;

output;

end;

end;

cards;

5.16 5.83 5.72

5.12 5.64 5.48

5.15 5.4 5.32

5.24 5.47 5.52

4.99 5.50 5.22

;

proc anova;

class treat rep;

model y=treat;

means treat/duncan;

means treat;

Run;

130**d ram feed weight ratio**

data ss;

do rep=1 to 5;

do treat=1 to 3;

input y@@;

output;

end;

end;

cards;

6.12 6.74 7.92

6.31 6.85 8.43

6.57 6.80 8.26

6.54 6.80 8.33

6.61 7.11 8.58

;

proc anova;

class treat rep;

model y=treat;

means treat/duncan;

means treat;

Run;

1-130**d ram feed weight ratio**

data ss;

do rep=1 to 4;

do treat=1 to 3;

input y@@;

output;

end;

end;

cards;

4.99 5.40 5.71

4.77 5.15 5.40

4.80 5.02 5.23

4.58 4.92 5.11

4.30 4.55 4.69

;

proc anova;

class treat rep;

model y=treat;

means treat/duncan;

means treat;

Run;
