# Supplementary material for: Effects of yeast culture on growth performance, immune function, antioxidant capacity and hormonal profile in Mongolian ram lambs
Source: Front Vet Sci. 2024 Jul 23;11:1424073. doi: 10.3389/fvets.2024.1424073 (PMC11301782; doi:10.3389/fvets.2024.1424073)
Supplement: Supplementary file 2 [file Data_Sheet_2.pdf]

	Groups	LZM (μg/mL)	ACP(U/L)	IL-1β (pg/mL)	IL-2 (pg/mL	IL-4 (pg/mL	IL-6 (pg/mL
Day 0	CON	0.71	33.14	19.29	232.09	8.104	112.569
		1.05	30.42	16.75	209.85	9.2424	115.6798
		1.43	24.79	13.97	177.45	11.201	127.398
		1.19	26.22	14.27	196.66	9.115	101.59
		0.83	32.48	17.81	203.41	9.268	118.88
		1.07	35.45	18.42	239.64	8.524	117.962
	YC	1.19	30.45	15.64	199.50	11.663	117.962
		1.07	33.42	18.91	210.34	10.667	134.328
		0.95	34.41	15.53	217.43	10.407	131.326
		1.10	33.14	17.34	216.87	10.9088	123.0018
		0.95	28.42	18.54	227.13	11.569	117.049
		1.31	39.03	18.05	229.92	10.238	114.344
	BG	1.34	37.05	17.10	203.41	8.889	123.557
		1.34	30.69	19.03	213.75	9.7682	120.755
		1.43	32.10	20.61	212.69	10.842	130.336
		0.95	35.62	21.01	222.25	11.757	120.734
		1.55	21.88	16.08	205.70	7.448	106.538
		1.43	26.82	20.34	224.68	9.905	122.61
Day 30	CON	1.07	51.61	19.10	229.76	10.335	144.021
		1.14	59.67	19.30	221.80	9.7352	132.9208
		1.19	50.46	18.34	216.75	10.569	139.578
		0.95	75.41	18.95	213.60	10.033	135.243
		1.31	71.56	19.69	242.35	9.09	124.866
		1.19	49.30	20.40	206.55	8.649	120.896
	YC	1.07	37.71	20.31	295.38	9.178	119.13
		0.95	51.83	22.15	305.75	7.896	136.316
		1.55	56.34	22.83	285.62	6.727	128.939
		1.24	51.68	21.08	282.47	7.5812	121.2568
		1.31	55.90	18.84	268.06	7.898	111.407
		1.31	56.61	21.29	257.56	6.207	110.492
	BG	0.83	61.51	19.41	236.55	7.219	108.146
		1.07	47.33	21.76	218.05	7.188	92.616
		1.10	62.77	19.93	227.26	8.137	101.5186
		1.43	69.86	21.62	230.05	11.046	106.283
		1.19	82.34	17.97	213.20	7.625	91.826
		0.95	52.82	18.89	238.47	7.607	108.722
Day 60	CON	1.31	41.11	21.93	279.28	8.716	159.114
		1.19	37.05	25.81	261.97	8.073	150.264
		1.29	49.10	23.19	246.88	9.1202	149.8328
		1.55	61.62	24.17	213.30	8.649	151.812
		1.07	58.70	20.02	220.07	9.678	134.958
		1.31	47.00	24.04	259.76	10.485	153.016
	YC	1.45	41.00	22.77	308.13	7.682	182.868
		1.48	47.25	25.13	305.31	6.6276	168.5288
		1.67	59.80	27.37	318.20	6.043	160.443
		1.07	40.34	28.92	301.97	6.485	170.678
		1.67	53.70	23.82	303.60	6.885	171.85
		1.55	41.39	22.77	294.63	6.043	156.805
	BG	1.67	41.11	24.01	257.00	8.09	156.931
		1.59	37.82	23.32	268.30	8.2528	161.5816
		1.79	34.52	20.74	273.15	8.522	153.235
		1.43	39.85	23.14	288.32	8.713	157.899
		1.55	34.08	25.48	266.42	9.777	157.782
		1.51	39.52	23.23	256.58	6.162	182.061
	CON	0.95	45.90	22.41	300.55	8.795	157.39
		1.55	48.76	20.15	254.60	8.039	150.533
		1.25	26.62	20.31	303.09	8.739	166.681

	CON	1.25	39.02	22.52	285.26	7.9258	156.3108
		1.31	34.79	23.46	264.49	7.052	154.269
		1.19	39.02	26.28	303.55	7.004	152.681
Day 90	YC	1.43	42.06	27.49	362.28	7.219	168.893
		1.60	54.65	26.67	348.40	6.696	174.96
		1.79	59.59	27.07	282.99	6.197	193.431
		1.43	54.56	26.28	365.43	6.697	192.252
		1.67	47.09	25.89	376.82	5.685	169.697
		1.67	69.93	26.60	354.49	7.682	150.527
	BG	1.07	42.41	29.51	314.65	7.53	155.436
		1.31	37.87	25.93	295.51	7.682	167.611
		1.36	41.36	25.88	305.31	7.7214	171.069
		1.55	44.29	22.93	290.09	7.215	174.869
		1.31	42.83	27.93	327.62	8.64	178.868
		1.55	39.40	23.10	298.67	7.54	178.561

IL-8 (pg/mL)	IL-10 (pg/mL)	IFN-γ (pg/mL	TNF-α (pg/mL)
19.593533	11.569	23.257	44.975
15.2462005	14.356	26.2314	46.5474
15.358521	17.553	33.99	53.922
15.13388	16.525	21.392	46.923
18.397344	15.002	31.737	41.301
17.201155	11.131	20.781	45.616
18.248169	12.015	25.942	63.162
19.906795	15.593	30.355	54.661
16.087856	11.645	28.789	44.975
17.6509405	12.5668	31.3606	53.8476
19.077482	13.108	37.312	55.406
19.214025	10.473	34.405	51.034
21.697307	13.642	36.316	53.189
17.988343	13.313	31.0136	52.3244
17.823855	16.619	29.043	42.492
18.152831	15.486	35.03	57.678
21.2553905	10.066	26.391	48.263
20.813474	10.752	28.288	60
21.869252	15.447	31.54	37.137
19.6331915	13.2214	28.6864	37.3668
18.506027	12.927	27.215	39.299
20.760356	14.194	27.681	37.425
21.2584435	12.549	28.46	38.045
20.647635	10.99	28.536	34.928
26.790295	12.917	37.103	67.793
28.119518	11.463	34.979	67.931
28.676266	14.143	33.728	62.702
27.0763525	13.0228	34.2756	60.5626
27.4549065	13.381	29.888	51.773
25.476439	13.21	35.68	52.614
26.433688	11.656	26.289	42.276
25.947071	12.067	30.68	56.556
25.6662895	13.123	29.1758	51.4114
26.542284	12.675	32.803	61.236
26.1903795	13.509	23.733	50.386
24.790295	15.708	32.374	46.603
42.103099	12.919	31.313	56.178
36.683163	11.508	34.691	52.276
39.397143	12.1956	32.4326	52.5202
37.000797	12.927	31.58	48.888
39.393131	10.871	29.888	54.196
41.793489	12.753	34.691	51.063
50.000001	12.181	51.666	63.038
48.9396125	10.6266	45.2592	66.0316
49.042834	9.136	45.513	65.719
48.836391	10.623	38.728	71.542
46.5657195	11.597	41.828	60.386
43.131438	9.596	48.561	69.473
37.40555	10.99	34.648	58.53
50.703677	12.9884	34.242	55.7944
48.448219	13.22	31.818	51.368
52.959135	14.013	31.54	59.917
35.778043	12.927	39.935	51.945
34.150536	13.792	33.269	57.212
40.09482	10.971	48.2	50.946
50.959864	11.451	43.811	73.34
53.645194	11.393	37.727	53.157

46.9980155	10.7758	40.283	57.9968
45.527342	10.605	37.575	55.049
40.350837	9.459	34.102	57.492
63.908027	9.794	45.223	59.815
56.68693	8.329	51.2996	70.0342
61.357252	8.901	47.725	75.691
52.016608	7.7	57.399	75.388
59.5034805	8.05	57.436	69.074
55.098934	7.2	48.715	70.203
47.6843	8.574	42.018	66.013
49.952821	8.461	39.898	66.637
50.0731885	8.7926	44.8332	62.9342
50.876601	8.674	46.423	59.634
48.8185605	8.338	56.863	56.947
49.269776	9.916	38.964	65.44

	Groups	IgG (g/L)	IgM (g/L)	sCD3 (ng/mL	sCD4 (ng/mL)	sCD8 (ng/mL
Day 0	CON	15.48	0.99	186.61	126.09	133.94
		20.93	0.91	294.84	197.38	175.06
		15.83	0.89	260.45	181.32	160.61
		12.49	0.75	263.79	191.78	156.71
		12.08	0.74	257.60	170.42	164.02
		18.16	1.05	299.39	220.94	173.35
	YC	14.12	0.90	238.50	161.28	133.87
		14.41	1.09	214.50	139.05	114.70
		12.37	0.81	240.45	167.03	152.55
		19.71	0.94	299.51	204.38	160.83
		12.04	0.75	200.86	126.35	107.08
		12.06	0.89	237.16	169.59	134.17
	BG	13.48	0.83	358.41	251.69	187.36
		13.82	0.77	289.62	196.58	148.76
		16.05	0.86	330.01	228.97	184.93
		14.54	0.76	229.16	119.16	112.34
		13.26	0.69	266.50	187.06	127.44
		11.77	0.74	264.03	196.05	131.73
Day 30	CON	18.92	1.36	332.20	179.20	171.58
		19.89	1.05	328.21	178.46	205.87
		17.93	1.26	388.06	201.01	176.47
		19.88	1.30	398.79	214.85	205.87
		20.77	1.23	496.79	283.30	230.98
		21.91	1.62	448.70	232.27	244.46
	YC	22.30	1.49	276.22	174.01	149.71
		22.45	1.78	246.39	115.60	115.21
		21.65	1.58	334.15	195.08	157.34
		21.67	1.69	345.38	199.86	158.50
		21.54	1.31	361.10	224.60	168.48
		20.29	1.63	441.67	261.35	194.80
	BG	21.57	0.95	342.22	216.52	221.44
		15.90	0.83	247.77	132.06	184.32
		19.02	0.92	268.50	143.72	139.66
		20.93	1.39	492.52	283.84	184.32
		19.95	1.09	362.21	200.48	184.20
		22.32	1.38	460.02	226.28	191.26
Day 60	CON	15.94	1.25	391.57	249.08	209.64
		16.28	1.29	308.70	200.82	193.40
		14.13	1.17	394.55	262.61	220.90
		16.01	1.24	483.39	285.47	226.53
		15.63	1.25	432.63	275.54	217.66
		17.66	1.29	338.56	220.94	189.68
	YC	19.65	1.69	447.33	188.94	188.49
		17.17	1.71	365.43	227.08	171.88
		19.13	1.66	491.70	299.46	244.39
		17.17	1.65	447.33	179.85	172.07
		17.92	1.67	447.33	240.55	206.88
		16.48	1.67	484.85	307.42	257.56
	BG	16.24	1.55	455.03	301.80	238.23
		17.17	1.48	408.39	248.65	196.31
		18.56	1.42	424.85	280.49	205.45
		15.52	1.46	401.82	242.37	211.59
		17.77	1.49	408.39	195.86	141.35
		17.77	1.48	351.86	222.72	184.93
	CON	15.70	1.36	437.72	222.68	211.02
		15.86	1.37	523.87	337.29	269.54
		15.90	1.36	437.72	276.21	275.57

Day 90	CON	15.87	1.34	448.97	276.21	238.53
		15.29	1.23	397.11	260.97	208.44
		16.59	1.37	448.44	283.92	228.08
	YC	23.21	1.73	512.06	289.48	217.32
		22.80	1.70	530.39	333.76	245.00
		22.27	1.70	594.28	354.21	260.65
		22.49	1.73	485.94	321.15	221.19
		23.25	1.67	547.61	355.10	264.00
		22.80	1.68	512.06	348.86	261.86
	BG	21.25	1.45	334.08	248.34	237.15
		21.97	1.46	406.40	281.49	226.25
		22.54	1.54	474.02	338.29	240.15
		20.55	1.35	475.52	303.23	237.44
		22.94	1.45	382.17	260.52	219.83
		22.60	1.49	366.22	257.07	196.67

sCD4/sCD8（ratio)
0.94
1.13
1.12
1.22
1.04
1.27
1.20
1.21
1.09
1.27
1.18
1.26
1.34
1.32
1.24
1.06
1.47
1.49
1.04
0.87
1.14
1.05
1.23
0.95
1.16
1.00
1.22
1.26
1.33
1.34
0.98
0.72
1.03
1.54
1.09
1.18
1.18
1.04
1.19
1.26
1.27
1.16
1.00
1.32
1.23
1.05
1.16
1.19
1.27
1.27
1.37
1.15
1.39
1.20
1.06
1.25
1.00

1.16
1.25
1.24
1.33
1.36
1.36
1.45
1.35
1.33
1.13
1.24
1.41
1.28
1.10
1.31

	Groups	MDA（nmol/r	SOD（U/mL)	CAT（U/mL	GSH-Px（U/	T-AOC（U/mL)
Day 0	CON	6.36	70.99	1.02	84.86	2.51
		6.83	66.44	3.17	76.20	2.22
		3.83	68.82	1.52	63.35	2.20
		5.11	73.02	1.75	76.20	2.20
		5.59	68.95	1.74	83.67	1.85
		2.93	64.71	1.26	72.91	2.22
	YC	5.54	75.21	2.30	74.10	2.01
		5.12	73.81	1.68	74.10	3.37
		2.99	69.39	1.23	77.69	2.41
		4.43	66.13	1.78	75.30	2.41
		3.56	62.68	1.78	76.49	2.55
		4.95	69.10	1.91	66.93	1.69
	BG	3.35	71.77	1.15	89.78	2.26
		3.35	63.62	1.15	77.87	1.27
		6.46	66.75	1.94	66.67	2.27
		4.64	61.26	1.44	74.67	2.27
		5.43	64.25	1.44	69.33	2.43
		4.64	72.87	1.52	88.89	3.12
Day 30	CON	3.18	71.96	2.72	78.81	2.78
		3.79	71.96	1.75	78.27	2.08
		2.96	70.20	1.86	94.03	2.61
		3.52	71.04	2.51	68.96	2.44
		3.46	69.93	2.42	69.85	2.69
		4.19	66.08	3.27	79.70	2.04
	YC	3.48	74.99	3.77	72.54	1.52
		2.81	75.81	3.95	77.37	1.52
		1.91	74.05	2.12	81.49	1.85
		2.81	73.69	2.23	89.55	1.78
		3.04	74.17	2.86	74.33	2.43
		2.81	71.57	2.25	68.96	1.56
	BG	2.24	66.82	2.67	67.16	3.08
		2.25	71.59	1.94	81.67	1.97
		3.93	70.97	2.30	78.81	2.34
		2.74	70.86	3.22	81.49	2.61
		2.81	72.51	2.53	111.04	2.92
		2.48	73.06	2.53	69.85	2.75
Day 60	CON	3.28	79.35	1.75	83.41	1.93
		1.45	73.47	1.57	88.85	1.48
		2.86	79.89	2.91	94.83	1.85
		2.64	80.07	1.47	88.95	1.81
		3.30	85.80	1.99	82.24	1.85
		2.32	80.78	2.25	94.83	1.92
	YC	2.45	69.16	5.18	98.18	3.89
		1.82	77.93	3.83	104.90	4.81
		3.31	78.87	3.09	91.47	3.54
		2.54	82.26	5.11	125.03	4.00
		2.43	83.53	4.09	100.70	4.32
		2.68	81.47	3.23	109.09	3.45
	BG	3.10	81.55	3.01	114.97	3.95
		1.47	66.18	3.09	109.09	5.00
		1.70	76.53	3.48	67.97	4.10
		2.64	76.12	3.61	123.36	4.10
		3.46	84.09	3.24	112.45	2.65
		3.46	74.68	3.01	126.71	4.81
	CON	2.17	72.73	2.62	122.43	1.69
		4.20	80.91	3.95	97.30	2.30
		5.72	77.89	4.77	81.08	1.73

Day 90	CON	3.82	79.82	4.08	94.86	1.96
		4.20	79.64	3.49	68.11	2.06
		2.80	76.36	2.04	120.00	2.01
	YC	3.38	79.38	4.58	115.14	1.81
		2.25	90.23	4.16	122.11	1.85
		3.38	83.05	4.22	137.84	1.81
		3.00	83.38	3.43	134.59	1.95
		3.46	78.11	3.60	109.46	2.01
		2.57	84.17	1.62	113.51	2.26
	BG	2.88	79.40	4.20	114.32	1.93
		2.48	83.70	6.34	107.35	2.26
		2.49	80.70	4.20	119.19	2.22
		2.43	83.06	2.20	87.57	2.10
		2.70	77.95	3.75	108.65	2.10
		1.62	79.39	1.83	107.03	2.01

	Groups	Cort（pg/	GH（nmol	T3（nmol/	T4（nmol/	INS（mU/	LEP（nmol/L)
Day 0	CON	1098.94	13.06	9.07	140.25	40.92	11.44
		1332.92	14.83	7.81	126.40	36.46	10.11
		1337.90	16.97	8.67	156.62	29.95	11.03
		1198.94	15.86	8.07	142.25	38.22	10.44
		1232.92	14.53	8.81	136.40	36.77	11.11
		1387.90	15.97	8.77	146.62	32.95	11.23
	YC	1062.80	17.92	9.13	158.05	44.08	10.79
		1293.10	16.15	7.32	163.33	37.93	11.68
		1480.98	15.62	8.37	174.54	46.58	13.68
		1162.80	16.92	9.03	168.53	43.81	11.79
		1273.10	16.75	7.92	161.53	38.93	10.68
		1380.98	15.96	8.17	164.36	45.58	12.68
	BG	1492.23	17.92	8.06	189.70	34.11	10.30
		1298.07	15.47	9.72	190.36	40.57	10.76
		1374.91	15.89	9.66	171.90	45.25	12.14
		1392.23	17.88	9.06	188.70	36.11	10.33
		1278.07	14.47	9.12	180.36	40.05	10.56
		1354.91	16.89	9.26	178.90	42.25	11.14
Day 30	CON	1442.45	12.56	5.54	160.69	41.25	15.05
		1254.56	13.52	6.53	180.47	42.09	15.64
		1205.64	10.11	4.53	170.94	37.93	14.25
		1342.45	12.16	5.64	168.69	41.03	15.49
		1354.56	11.52	6.13	177.47	40.09	15.42
		1275.64	12.11	4.93	171.94	38.93	14.54
	YC	665.82	16.21	10.12	125.75	27.91	9.66
		680.75	18.88	8.67	104.65	22.97	10.94
		824.26	21.39	8.09	131.02	24.80	10.89
		675.82	16.41	10.02	122.75	27.05	9.96
		660.75	17.88	9.67	114.65	23.97	10.39
		724.26	20.39	9.09	128.02	24.51	10.30
	BG	660.84	17.72	5.76	222.67	32.44	10.62
		551.31	14.83	4.53	206.62	30.07	11.50
		805.21	15.82	6.46	204.21	36.43	9.52
		760.84	17.52	5.56	212.67	31.44	10.19
		651.31	14.93	4.87	216.62	32.07	11.06
		705.21	15.62	6.17	209.21	33.43	9.92
Day 60	CON	1064.09	14.05	9.63	162.01	42.75	11.90
		994.39	15.39	7.20	179.15	45.08	12.73
		1108.89	17.13	7.97	189.81	36.60	10.62
		1014.09	15.05	8.63	172.01	43.75	11.76
		1094.39	15.59	7.82	169.15	44.08	12.29
		1101.89	16.13	7.87	180.81	39.60	10.90
	YC	705.64	17.91	8.46	176.51	27.46	13.36
		840.06	20.21	9.47	183.77	30.43	14.68
		536.38	16.08	7.73	173.77	26.62	13.13
		735.64	18.91	8.86	174.51	27.55	13.59
		740.06	19.21	8.47	177.77	29.43	14.32
		636.38	17.08	7.83	175.77	28.62	13.60
	BG	1362.13	9.72	9.20	156.73	38.57	14.85
		1487.25	9.72	10.30	134.98	30.45	15.64
		1279.89	11.54	10.06	142.89	35.27	15.51
		1332.13	10.42	9.90	146.73	36.57	14.47
		1387.25	10.82	10.10	154.98	32.45	15.22
		1299.89	10.54	10.26	148.89	33.27	15.05
		1452.40	11.49	10.12	121.79	31.14	12.81
		1330.53	10.18	10.12	136.95	30.12	12.81
	CON	1396.98	13.05	8.90	111.24	38.26	10.06

Day 90	CON	1352.40	11.89	10.02	118.79	32.14	12.11
		1380.53	10.98	10.32	126.95	33.12	12.09
		1326.98	12.05	9.70	121.24	36.26	10.57
	YC	994.39	10.96	6.49	157.39	29.90	9.61
		700.66	10.50	5.20	148.82	21.14	10.80
		966.48	9.32	5.91	114.54	27.60	12.00
		894.39	11.16	6.09	137.39	29.01	9.89
		810.66	10.20	5.50	128.82	23.14	10.02
		796.48	10.32	5.31	124.54	25.60	11.29
	BG	1377.73	14.05	7.08	122.45	30.78	13.86
		1106.94	14.90	6.31	138.93	31.97	11.21
		1397.64	14.64	9.53	124.43	38.43	12.63
		1277.73	13.85	7.88	120.45	31.78	13.13
		1206.94	14.70	6.91	136.93	32.97	11.64
		1197.64	14.24	9.13	126.43	36.43	12.28
